# Prognostic Impact of CEACAM1 in Node-Negative Ovarian Cancer Patients

**DOI:** 10.1155/2018/6714287

**Published:** 2018-06-28

**Authors:** Leticia Oliveira-Ferrer, Roshni Goswami, Vladimir Galatenko, Yi Ding, Kathrin Eylmann, Karen Legler, Sascha Kürti, Barbara Schmalfeldt, Karin Milde-Langosch

**Affiliations:** ^1^Department of Gynecology, University Medical Center Hamburg-Eppendorf, Martinistraße 52, 20246 Hamburg, Germany; ^2^Moscow State University, Moscow, Russia

## Abstract

The underlying mechanisms of ovarian cancer (OvCa) dissemination are still poorly understood, and novel molecular markers for this cancer type are urgently needed. In search of adhesion molecules with prognostic relevance in OvCa, we compared tumors with good outcome (alive > 3 years) and those with poor outcome (dead < 2 years) within data from The Cancer Genome Atlas (TCGA). The carcinoembryonic antigen-related cell adhesion molecule 1 (CEACAM1) turned out as the only gene with differential expression in these groups. In order to further investigation on its role in OvCa, we analyzed CEACAM1 mRNA levels extracted from TCGA microarray data (*n* = 517) as well as CEACAM1 protein expression by Western blot analysis in a cohort of 242 tumor samples. Further, CEACAM1 localization in tumour tissue was evaluated by immunohistochemistry and CEACAM1 splice variants by RT-PCR in representative tumours. In Kaplan–Meier analysis, high CEACAM1 mRNA levels significantly correlated with longer survival (*p* = 0.008). By Western blot analysis in the second cohort, similar associations of high CEACAM1 protein levels with longer recurrence-free survival (RFS, *p* = 0.035) and overall survival (OAS, *p* = 0.004) were observed. In multivariate Cox regression analysis including clinical prognostic parameters, CEACAM1 mRNA or protein expression turned out as independent prognostic markers. Stratified survival analysis showed that high CEACAM1 protein expression was prognostic in node-negative tumors (*p* = 0.045 and *p* = 0.0002 for DFS and OAS) but lost prognostic significance in node-positive carcinomas. Similarly, high CEACAM1 mRNA expression did not show prognostic relevance in tumors with lymphatic invasion (L1) but was associated with longer survival in cases without lymphovascular involvement. Further analysis showed a predominance of 4S and 4L isoforms and mostly membraneous CEACAM1 localization in ovarian tumours. Our results suggest that CEACAM1 might be an independent favorable prognostic marker in OvCa, especially in the subgroup of patients with solely intraperitoneal metastasis.

## 1. Introduction

Epithelial ovarian carcinoma (EOC) is the gynecologic tumor with the highest mortality. Since it is asymptomatic in early development, it is mostly diagnosed in advanced stages when tumor dissemination has already taken place. Tumor spread occurs either intraperitoneally, without the nodal involvement and/or through lymphatics, giving rise to retroperitoneal metastatic lesions. Up to now, the biologic background of these two different metastatic routes is poorly understood. Independently of the dissemination mode, ovarian cancer therapy includes optimal surgical tumor reduction (debulking) followed by platin-based combination chemotherapy. In spite of intensive research, there are no established molecular prognostic or predictive markers for this cancer type, and new molecular targets for an individualized therapy are urgently needed.

The carcinoembryonic antigen-related cell adhesion molecule 1 (CEACAM1) is a member of the carcinoembryonic antigen (CEA) family and belongs to the immunoglobulin superfamily. This glycoprotein can bind homophilically as well as heterophilically to the other CEA family members [1]. Currently, 12 alternative splicing forms of the CEACAM1 gene are known [2, 3], differing in the number of extracellular immunoglobulin-like domains, the membrane anchorage and the length of the cytoplasmic domain [4]. Among them, only four isoforms are expressed at mRNA level: CEACAM1-4L, CEACAM1-4S, CEACAM1-3L, and CEACAM1-3S, including 4 and 3 heavily glycosylated extracellular domains and a long (L) or short (S) cytoplasmic tail, respectively [2]. CEACAM1 is expressed in the epithelia and leukocytes in addition to the endothelia of newly formed vessels and exerts very different biological functions such as immune response regulation, neovascularization or insulin clearance [5].

The role of CEACAM1 in cancer strongly differs depending on the origin of the tumor cell. Downregulation of CEACAM1 has been described in prostate, colon, and breast cancer, whereas CEACAM1 upregulation correlates with disease progression in melanoma and pulmonary adenocarcinoma [6–11]. Here, CEACAM1 is involved in several cellular functions such as proliferation, apoptosis, angiogenesis, invasion, and migration [12]. In selected tumor types like melanoma and glioma, first experiments indicate that CEACAM1 might be a suitable target for immunotherapy [13, 14]. The majority of the mentioned studies analyzed total CEACAM1 levels. Recently, the specific role of concrete isoforms has been studied by different groups in melanoma and colon cancer [15–17].

In ovarian cancer, Li et al. have recently shown a membrane-associated CEACAM1 staining in primary low-grade adenocarcinomas, whereas in high-grade adenocarcinomas and metastatic lesions CEACAM1 were mainly localized in the cytoplasm. These data suggest a tumor suppressor function of membranous CEACAM1, while cytoplasmic CEACAM1 might be involved in tumor progression and metastasis [18].

In order to deeply evaluate the relevance of CEACAM1 for ovarian cancer progression, we analyzed its predictive and prognostic value at both the mRNA and protein level in two well-characterized ovarian cancer cohorts.

## 2. Material and Methods

### 2.1. Patients

For Western blot analysis, a total of 242 patients with epithelial ovarian tumors and primary surgery at the University Medical Centre Hamburg-Eppendorf between 1997 and 2012 were included. Patients gave their written approvals for examining tissue samples and reviewing their medical records according to our investigational review board and ethics committee guidelines (number 190504). The median follow-up time for patients with primary cancer (*n* = 210) was 21 months. Clinical outcome of all patients was followed from date of surgery until December 2016. Detailed patient characteristics are listed in the Supplementary Table S1. For comparison, samples from four benign ovarian cystadenomas, 16 tumors of low malignant potential (LMP, borderline tumors), and 12 recurrent carcinomas were analyzed.

### 2.2. Western Blot Analysis

Western blot analysis was performed as described previously [19, 20]. Briefly, equal amounts of protein (20 *μ*g) of each sample were loaded per well. The protein lysate from the cell line OAW42 was used as a reference and internal control in all blots. After electrophoresis and blotting to PVDF membranes, CEACAM1 was detected using anti-human CEACAM-1/CD66a antibody (AF2244, R&D Systems, Minneapolis, USA) antibody. Membranes were blocked 1 hour at room temperature with 1% blocking solution containing 0.1 M maleic acid and 0.15 m NaCl pH 7.5 in TBST and subsequently incubated with the antibody (0.2 *μ*g/ml in 0.1% blocking/TBST solution) overnight at 4°C. Secondary antibody (mouse anti Goat, sc-2354, Santa Cruz Biotechnology Inc., Heidelberg, Germany) was also diluted in 0.1% blocking solution and incubation was performed for 1 hour at room temperature. Equal loading was verified by immunoblotting with *β*-actin antibody (sc-47778, Santa Cruz Biotechnology). After visualization by chemiluminescence reagent (SuperSignal West Pico kit, Pierce, Rockford, IL, USA), band intensities were quantified by densitometry (Imaging Densitometer GS-700, Bio-Rad, Munich, Germany) and calculated as percent intensity of the specific control sample.

### 2.3. Immunohistochemistry

Immunohistochemical analyses were performed as previously described [19]. Briefly, for the detection of CEACAM1 on an ovarian cancer tissue, slides of 4 *μ*m were deparaffinized, microwaved in citrate buffer pH 6, and incubated overnight at 4°C with the CD66a polyclonal antibody (R&D Systems, Minnesota, USA; concentration: 2.5 *μ*g/ml). For detection, slides were incubated with biotin-labelled anti-goat immunoglobulin (IgG), preformed ABC-Complex (Vectastain, Vector Laboratories) and DAB-substrate kit (Vectastain, Vector Laboratories). All slides were counterstained with haematoxylin. As negative controls, normal goat immunoglobulin (Dako Denmarck A/S, Glostrup, Denmark) was used instead of primary antibody. Images were performed using an AxioVision40 Microscope (Carl Zeiss Imaging Solutions).

### 2.4. Isoform-Specific RT-PCR

RNA extraction and quality analyses were performed as mentioned before [21]. 1 *μ*g RNA was reverse transcribed using the Transcriptor First-Strand Synthesis Kit (Roche). The PCR were performed by ALLin™ Hot Start Taq Polymerase Kit (highQu, Kraichtal, Germany) in a total volume of 25 *μ*l containing 1 *μ*l of first-strand cDNA solution, 0.5 *μ*l of ALLin Hot Start Taq Polymerase (5 u/*μ*l), 5 *μ*l of 5X-ALLin PCR Buffer, 2 *μ*l each of the PCR primers (10 pmol/*μ*l), and 14.5 *μ*l H_2_O. The reactions were initiated by heating the samples to 95°C for 60 s, followed by 40 cycles at 95°C for 15 s, 55–65°C for 15 s, and 72°C for 15 s and an extension at 4°C for 10 min. The products were analyzed on 2% agarose gels in Tris-borate-EDTA buffer and visualized by ethidium bromide staining. The sense (forward (FP)) primer is common for the two isoforms, whereas the antisense (backward (BP)) primers are selective for the L or the S isoform, respectively. Following oligonucleotide, primers were used in order to amplify the different isoforms as previously described [22]: humanCEACAM1_FP49: 5′-GCAACAGGACCACAGTCAAGACGA-3′, humanCEACAM1_BP60: 5′-GTGGTTGGAGACTGAGGGTTTG-3′ and humanCEACAM1_BP59: 5′-TGGAGTGGTCCTGAGCTGCCG-3′. For quantification of CEACAM1-4L/4S ratio, a triple-primer PCR procedure was used as previously described [22].

### 2.5. Gene Expression Analysis and Statistics

Gene expression data from serous ovarian adenocarcinomas were retrieved from The Cancer Genome Atlas (TCGA) Research Network. Patient annotation data and CEL files with scans of microarrays (microarray type Affymetrix HT HG U133A) were downloaded from TCGA [23] and jointly preprocessed in Affymetrix Expression Console using the robust multiarray average (RMA) method [24]. In order to identify prognostic genes, a standard differential expression analysis was applied to the group of patients with survival less time than two years versus the group of patient with vital status “alive” and follow-up of at least three years. The analysis was limited to patients with stages III and IV, and the groups contained 97 and 71 patients, respectively. The differential expression analysis was performed in Affymetrix Transcriptome Analysis Console. As the compared groups were similar, relatively liberal thresholds were used, namely, 1.5x for fold change and 0.05 for *p* value. Statistical analyses were performed using the Statistical Package for Social Sciences (SPSS) program (SPSS Inc., Chicago, IL, USA), version 15.

For statistical analysis of CEACAM1 protein expression levels in primary carcinomas, all tumor cases were divided into two groups of equal size (</> median), representing low and high protein expression. Chi-square tests were used to examine the correlations between CEACAM1 and clinicopathologic factors (age, FIGO stage, histology subtype, grading, and residual postoperative tumor). For prognostic parameters, the following groups were compared: histological grading (G1/G2 versus G3), FIGO stage (I/II versus III versus IV), histological subtype (serous versus others), and residual tumor (none versus >1 cm). Survival curves were plotted by Kaplan–Meier analysis. Differences between survival curves were evaluated by log-rank tests. Probability values (*p* value) ≤ 0.05 were considered statistically significant.

## 3. Results

### 3.1. CEACAM1 mRNA Levels in Ovarian Carcinomas (TCGA Cohort)

Since we were interested in the role of adhesion proteins in ovarian cancer progression, we decided to analyze the microarray data of the TCGA cohort. For this purpose, 536 CEL files were downloaded. We excluded 16 duplicates and 3 cases with missing follow-up data, resulting in a cohort of 517 cases. The characteristics of this cohort are given in the Supplementary Table S2.

In the first screening approach, we compared stage III/IV cases with relatively good outcome (alive > 3 years) and those with poor outcome (dead < 2 years). Sixty-two probesets passed the thresholds, including only one adhesion molecule, CEACAM1 (Affymetrix number 209498_at), with a 1.5fold higher expression in tumors with a better prognosis. This led us to a more intensive analysis of CEACAM1 expression in this cohort.

Among the expression data generated by Affymetrix chips, there are five probesets for CEACAM1 (Affymetrix number 209498_at; 211883_x_at; 2118899_x_at; 210610_at; 206576_s_at). The mean expression levels in this cohort were relatively low for all probesets, with highly significant correlations among each other (not shown). Therefore, we concentrated on the probeset 209498_at with differential expression between the two groups and the highest expression level among the probesets (mean expression 23.2; median 12.4; range 5 to 327). The sequences of the probes included in this probeset allow for the detection of all CEACAM1 mRNA variants (Affymetrix).

For further analysis, the cohort was divided into two groups with low or high CEACAM1 mRNA levels with the median as the cutoff value. By Chi-square tests, these groups were compared with respect to the known tumor parameters. There was no significant association of CEACAM1 expression with age at diagnosis, grading, clinical stage, residual disease after surgery, and lymphovascular invasion and vascular invasion (Supplementary Table S3).

**Figure 1:**
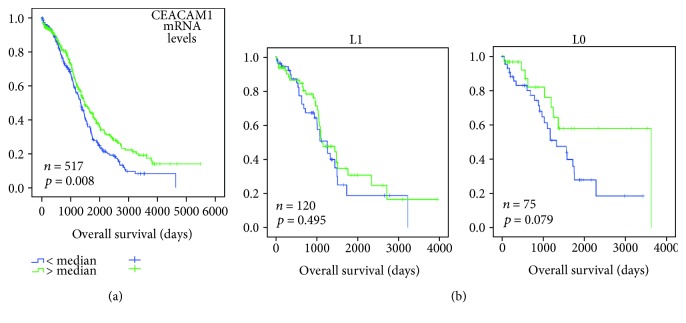
CEACAM1 mRNA levels in ovarian carcinomas (TCGA cohort). (a) Kaplan–Meier analysis showing a significant correlation of high CEACAM1 mRNA levels with longer overall survival. (b) Kaplan–Meier analysis and log-rank tests stratified for tumors with (L1) and without (L0) lymphovascular invasion. High CEACAM1 mRNA levels showed a clear association (*p* = 0.079) with shorter overall survival only in patients without lymphovascular invasion (L0) in comparison with those with lymphovascular invasion (L1).

In survival analysis, we could show a significantly longer survival in carcinomas with high CEACAM1 mRNA levels (*p* = 0.008; Figure 1(a)). In stratified Kaplan–Meier analysis regarding the presence of lymphovascular invasion, CEACAM1 was weakly prognostic in L0 tumors (*p* = 0.079) but not in L1 carcinomas (Figure 1(b)), whereas no difference in tumors with and without vascular invasion was observed (not shown).

In multivariate Cox regression analysis also including clinical stage, residual disease after surgery and histological grading, high CEACAM1 mRNA levels (>median) turned out as an independent and significant prognostic indicator with a hazard ratio of 0.75 (95% CI 0.59–0.95; *p* = 0.019; Table 1).

### 3.2. CEACAM1 Protein Expression in Ovarian Tumor Samples

**Figure 2:**
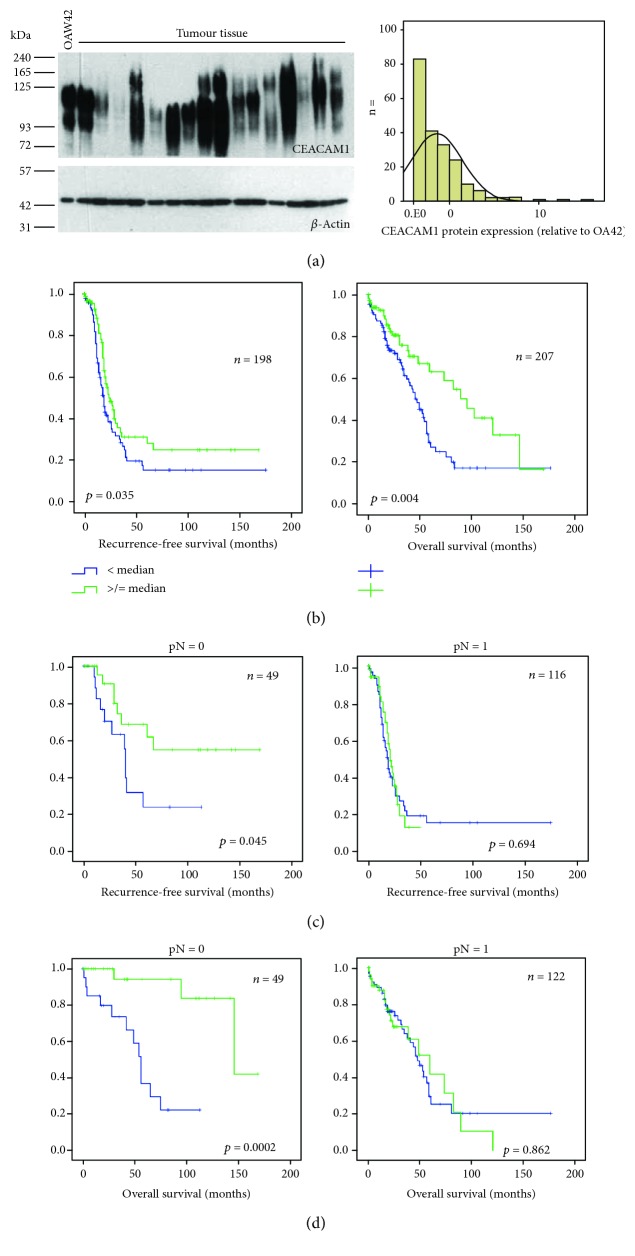
CEACAM1 protein expression in clinical tumor tissue samples. (a) Representative Western blot results of CEACAM1 expression in ovarian carcinomas (left) and distribution of the relative expression within the cohort (right). Protein extract from the ovarian cancer cell lines OAW42 was included in each gel as an internal control. (b) Kaplan–Meier analysis showing correlations of high CEACAM1 protein expression with longer recurrence-free survival and overall survival. (c, d) Kaplan–Meier analysis and log-rank tests, stratified for tumors with (pN = 1) and without (pN = 0) nodal involvement. High CEACAM1 expression correlated significantly with shorter recurrence-free survival and overall survival only in patients without nodal involvement.

In order to validate the prognostic significance of CEACAM1 expression on a protein level, Western blot analysis was performed in tissue samples from our clinic, including 210 primary ovarian carcinomas, 12 recurrent tumors, 16 borderline tumors, and 4 cystadenomas. Due to a variety of splice variants and glycosylation variants, the size of the detected CEACAM1 bands ranged from 70 to 200 kDa, with strong variations in band intensity (Figure 2(a)). The different variants did not always appear as clearly distinguishable bands in Western blots, but in many cases, it led to a continuous smear. For densitometry, all CEACAM1 bands were therefore combined, and band intensities were calculated relative to the positive control cell line OAW42 which was set as 1 and corrected for equal actin loading.

Regarding tumors of different malignancy, no significant differences in CEACAM1 expression between cystadenomas, borderline tumors, and invasive carcinomas were found. In addition, expression did not differ between primary and recurrent carcinomas (not shown).

Regarding the primary ovarian carcinoma samples, all cases were divided into two groups with CEACAM1 expression below/above the median value for statistical analysis. Using these groups, we found significant correlations of high CEACAM1 expression with the presence of distant metastasis, but no significant associations with grading, residual tumor after surgery, and lymph node involvement. Regarding FIGO stage, significantly higher CEACAM1 levels were detected in early stages (FIGO I-IIIb) and cases with distant metastasis (FIGO IV) in comparison to the most frequent stage IIIc tumors (Table 2). Pearson correlation did not reveal any association of CEACAM1 expression with age at diagnosis or CA125 serum levels before surgery (not shown).

Kaplan–Meier analysis revealed a significant association of high CEACAM1 levels with a longer recurrence-free survival (*p* = 0.035) and overall survival (*p* = 0.004; Figure 2(b)) indicating that CEACAM1 might function as a tumor suppressor in ovarian carcinomas. Hazard ratios in tumors with high CEACAM1 expression were 0.516 (95% CI 0.326–0.817; *p* = 0.005) for death and 0.669 (95% CI 0.457–0.980; *p* = 0.039) for recurrence.

In stratified Kaplan–Meier analysis regarding the lymph node involvement, high CEACAM1 expression was strongly prognostic of longer RFS and OAS in node-negative tumors, whereas it lost prognostic significance in node-positive carcinomas (Figure 2(c) and 2(d)). In multivariate Cox regression analysis including clinical stage, residual tumor after surgery, and nodal involvement, CEACAM1 remained a prognostic indicator for overall survival but lost its significance for recurrence-free survival (Table 3).

### 3.3. CEACAM1 Localization in Ovarian Cancer by Immunohistochemistry

**Figure 3:**
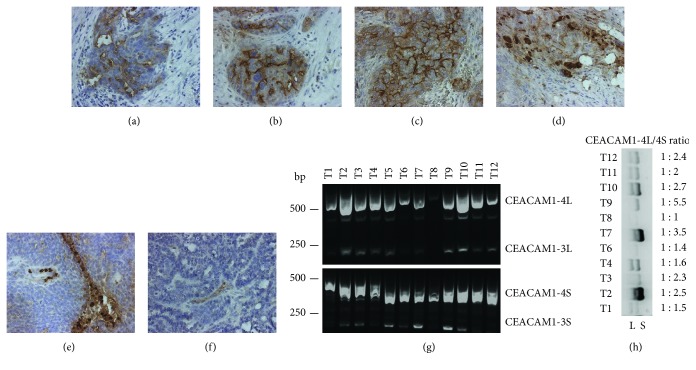
CEACAM1 protein localization and mRNA isoforms in selected ovarian tumors as detected by immunohistochemistry (a–f) and RT-PCR (g, h). CEACAM1 expression was observed in tumor cells as a mixed pattern of strong membranous staining, mainly within lumen-like structures and a weak cytoplasmic reactivity (a–c). Occasionally, a strong cytoplasmic CEACAM1 expression was observed in single cells or small cell aggregates (d). CEACAM1 staining was also found in lymphocytes (e) and endothelial cells from diverse small tumor-associated capillaries (f). RT-PCR of RNA isolated from ovarian cancer tissue using the oligonucleotides FP49 and BP60 (L-isoforms, upper panel) and FP49 and BP60 (S-isoforms, lower panel). Mainly products corresponding to the 4 (4L and 4S) isoform were amplified, whereas products corresponding to the 3 isoform were very weakly or not detected (g). A triple-primer RT-PCR was performed to quantify the expression ratios of the L and S splice isoforms. In all analyzed tumor tissues, the S isoform was expressed to a higher extent than the L isoform (h).

In order to validate that CEACAM1 protein is expressed in tumor cells, we performed immunohistochemistry with formalin-fixed, paraffin-embedded tumor samples (*n* = 15) from the Western blot cohort. Regarding tumor cells, different staining patterns were observed: The most frequent type included membranous CEACAM1 staining, mainly within lumen-like structures of the tumor, partly accompanied by weak cytoplasmic reactivity (Figures 3(a)–3(c)). In some cases, there was a strong cytoplasmic staining in <20% of the tumor cells (Figure 3(d)), whereas in other cases, a weak or no CEACAM1 expression within tumor cells was detectable (Figures 3(e) and 3(f)). Regarding the stromal components, CEACAM1 staining was frequently found in lymphocytes (Figure 3(e)) and vessel walls (Figure 3(f)).

### 3.4. mRNA Levels of CEACAM1 Isoforms in Ovarian Cancer

The mRNA levels of the four isoforms CEACAM1-4L/-4S/-3L and -3S were determined by RT-PCR using specific primers that discriminate between these splice variants as previously described [22]. Here, the forward primer is common for the two isoforms CEACAM1-4 and -3, whereas the backward primers are specific for the L or the S isoforms, respectively. Using this PCR procedure in a representative group of 13 samples, we found that ovarian cancer tissue expresses primarily both 4L and the 4S isoforms of CEACAM1, whereas a very weak or no detection of the CEACAM1-3L and -3S variants was observed (Figure 3(g)). A triple-primer RT-PCR was performed to quantify the expression ratios of the L and S splice isoforms. In almost all analyzed tumor tissue samples, the S isoform was stronger expressed than the L isoform with a S : L ratio from 1 to 5.5 (3 h).

## 4. Discussion

CEACAM1 is an immunoglobulin-like cell adhesion molecule with a broad range of biological functions that has been frequently described to play a key role during tumor progression in diverse tumor entities. Depending on the origin of the tumor, CEACAM1 might differently affect the tumorigenic process by acting as a tumor suppressor gene or as a metastatic driver. The present data show for the first time a significant association of CEACAM1 expression with disease outcome in ovarian cancer. This could be shown in two independent large cohorts on mRNA (TCGA data) and protein level (Hamburg cohort).

CEACAM1 has been found to be normally expressed in diverse epithelia such as in the colon, gallbladder, pancreas, kidney, prostate, and endometrium, frequently showing a characteristic apical membranous staining [25]. In contrast, in normal fallopian tube epithelium, which is considered to be the precursor tissue of most high-grade ovarian carcinomas, CEACAM1 expression was not detectable [26]. Regarding CEACAM1 deregulation during tumor progression, CEACAM1 is considered to be downregulated in breast, colon, endometrium, prostate, and hepatocellular carcinomas, suggesting a tumor suppressor role of this molecule, but it is upregulated in gastric carcinoma, squamous cell carcinoma of the lung, or melanoma [27]. Here, increased expression frequently correlates with metastasis and poor prognosis [10, 28, 29]. Our results show a strong CEACAM1 expression in most ovarian cancer tissue samples, whereas its impact on prognosis points to a tumor suppressor function. High CEACAM1 expression significantly correlates with longer recurrence-free and overall survival as shown at mRNA and protein levels in two independent cohorts comprising material from 517 and 210 ovarian cancer patients, respectively.

Controversial findings have also been reported about CEACAM1 function in tumor biology. As a tumor suppressor, its loss promotes early tumor development by reducing CEACAM1-mediated growth inhibitory signaling [30]. The N-terminal domain of CEACAM1, essential for intercellular adhesion, was not necessary for this tumor inhibitory effect. An antiproliferative effect of CEACAM1 has also been shown in preclinical models in bladder, prostate, and colon cancer cell lines [31–34]. On the other hand, CEACAM1 has been described as a driver of invasion and metastasis in colorectal cancer and hepatocellular carcinoma [35, 36]. Our data on two independent OvCa cohorts indicate that high CEACAM1 levels exert a tumor suppressor function in ovarian cancer, possibly by reducing tumor cell proliferation as previously described in other entities. In contrast, the described proinvasive and promigratory function of CEACAM1 seems to be not as relevant in ovarian cancer. Indeed, for intraperitoneal metastasis, which is the main route of tumor spread in those patients without nodal involvement, the key features for tumor progression are tumor cell growth, detachment, and immune evasion rather than cell motility and invasive potential.

Another functional aspect is the role of CEACAM1 as an immune modulator. In the context of cancer, there are contradictory reports describing CEACAM1 as an activator or repressor of the immune response [13]. Recently, a CEACAM1-associated decrease of STAT3 activity and CCL2 secretion was found in colorectal carcinoma, thus regulating inflammatory signalling networks and decreasing metastatic burden [37]. Further, CEACAM1-3S enhanced immunogenicity of melanoma cells by cell surface upregulation of NKG2D receptor ligands, thereby sensitizing them to lysis by natural killer cells [15]. In line with these studies, our data suggest that CEACAM1 overexpression might increase OvCa cell immunogenicity, thus decreasing their tumorigenicity.

Tumor spread in ovarian cancer occurs either intraperitoneally or through lymphatics, giving rise to retroperitoneal metastatic lesions. Remarkably, the correlation between high CEACAM1 protein levels and longer recurrence-free or overall survival was highly significant only within the subpopulation of ovarian cancer patients with solely intraperitoneal metastasis without nodal involvement. In contrast, CEACAM1 does not show any prognostic significance in tumours with nodal involvement in our Western blot cohort. The mRNA results obtained using the TCGA database which includes information about lymphovascular involvement, but not about nodal metastasis, point to the same direction. Thus, CEACAM1 expression might have an inhibitory impact on intraperitoneal metastasis but obviously loses this effect if lymphatic involvement takes place. The fact that a significant prognostic impact is shown in the entire cohort (*n* = 517), but only a borderline significance in the L0 and no impact in the L1 subcohorts can be explained by the relatively low case numbers with information about this point (*n* = 120/*n* = 75 for L1/L0; Table S2).

Our data on the prognostic impact of CEACAM1 expression support the theory that these two modes of OvCa progression represent different tumor types which rely on different biologic backgrounds. Recent studies have shown that the tumor cell differentiation status (epithelial-mesenchymal transition) might influence the route of metastasis in ovarian cancer [38]. Accordingly, we could demonstrate that a 85 kDa fragment of the epithelial marker E-Cadherin is associated with intraperitoneal metastasis, whereas VEGF-C and VEGF-D are more highly expressed in tumors displaying retroperitoneal tumor spread [39, 40]. Although CEACAM1 expression does not correlate with the mode of OvCa metastasis, its relevance for patient outcome strongly differs between both ovarian cancer types.

Originally, most studies analyzed total CEACAM1 expression without taking into consideration the prevalence and specific role of certain isoforms. Indeed, recent results demonstrate an isoform-specific functionality of CEACAM1, thus increasing the complexity of this system. In malignant melanoma cell lines, CEACAM1-3S, CEACAM1-3L, CEACAM1-4S, and CEACAM1-4L affected migration, invasion, and immunogenicity in an isoform-specific manner [15]. Additionally, isoform-specific cellular localizations could be shown: CEACAM1-4 variants were mainly membrane associated, whereas CEACAM1-3 isoforms were predominantly localized intracellularly [15]. In our analysis of these isoforms in OvCa tumor tissue samples, we found high mRNA levels for CEACAM1-4L and -4S, whereas CEACAM1-3L and 3S were only weakly or not detectable. In line with prior results [15], we observed a primarily membranous CEACAM1 staining in most analyzed ovarian cancer samples together with a weak cytoplasmic expression. Further, in a small group of ovarian cancer samples, higher levels of the 4S isoform compared to the long variant 4L were found. The CEACAM1-4S isoform has been shown in breast cancer to inhibit tumor cell invasion and migration and to promote apoptosis [41, 42]. Further functional experiments would be necessary to specifically clarify the impact of both isoforms in ovarian cancer progression.

## 5. Conclusion

To our knowledge, this is the first study reporting a significant association of high CEACAM1 expression with a better outcome of ovarian cancer patients in two independent cohorts. This prognostic impact is restricted to tumors with intraperitoneal metastasis but without lymph node involvement. Thus, CEACAM1 might be an independent favorable prognostic or predictive marker for node-negative ovarian cancer patients.

## Figures and Tables

**Table 1:** Multivariate Cox regression analysis including CEACAM1 RNA levels.

Overall survival				
Variables in multivariate analysis		*p*	HR	95% CI
CEACAM1 mRNA level	>median	0.019	0.75	0.59–0.95

Clinical stage	I-II	0.136		
	III	0.183	1.65	0.79–3.44
	IV	0.007	2.05	0.94–4.43

Tumor grading	G3	0.127	1.32	0.92–1.89

Residual tumour after surgery	No macroscopic tumour	0.0002		
	1–10 mm	0.001	1.87	1.31–2.68
	11–20 mm	0.004	2.26	1.31–3.92
	>20 mm	0.00001	2.51	1.67–3.77

**Table 2:** Correlations of CEACAM1 protein expression with clinical and histological tumor parameters (missing values to *n* = 210: no information).

		Low CEACAM1 expression	High CEACAM1 expression	
		<median	>median	*p*
Clinical stage	FIGO Ia–IIIb	4	14	
	FIGO IIIc–IV	97	86	**0.013**

Grading	G1-2	29	26	
	G3	75	74	0.762

Lymph node involvement	N0	21	28	
	N1	67	57	0.185

Distant metastasis	M0	79	63	
	M1	16	27	**0.034**

Residual tumor after surgery	No macroscopically visible tumor	67	71	
	>1 cm	24	14	
	>1 cm	13	11	0.273

**Table 3:** Multivariate Cox regression analysis including CEACAM1 protein expression.

Overall survival				
Variables in multivariate analysis		*p*	HR	95% CI
CEACAM1 expression </> median		0.002	0.44	0.26–0.74
FIGO stage	Ia–IIIb	0.0001		
	IIIc	0.238	1.81	0.67–4.88
	IV	0.002	5.15	1.87–14.21
Residual tumor after surgery	No macroscopic tumor	0.0004		
	<1 cm	0.002	2.33	1.37–3.97
	>1 cm	0.002	2.90	1.47–5.71

Recurrence-free survival				
CEACAM1 expression </> median		0.291	0.80	0.53–1.21
FIGO stage	Ia–IIIb	0.001		
	IIIc	0.005	4.55	1.59–13.04
	IV	0.0003	7.67	2.55–23.05
Residual tumor after surgery	No macroscopic tumor	0.0001		
	<1 cm	0.005	2.04	1.25–3.34
	>1 cm	0.0001	3.60	1.89–6.88

## Data Availability

The mRNA data used to support the findings of this study and corresponding clinical and histological information can be downloaded from The Cancer Genome Atlas (TCGA) Research Network [23]. The exact Western blot data used to support the findings of this study are available from the corresponding author upon request.

## Supplementary Materials

Supplementary Materials Table S1: cohort of ovarian cancer patients used for Western blot analysis. Table S2: TCGA cohort (*n* = 517) used for analysis of mRNA data. Table S3: correlations of CEACAM1 mRNA levels with clinical and histological tumour parameters.
